# Artifact Correction in Short-Term HRV during Strenuous Physical Exercise

**DOI:** 10.3390/s20216372

**Published:** 2020-11-08

**Authors:** Aleksandra Królak, Tomasz Wiktorski, Magnus Friestad Bjørkavoll-Bergseth, Stein Ørn

**Affiliations:** 1 Institute of Electronics, Lodz University of Technology, 93-005 Lodz, Poland; 2 Department of Electrical Engineering and Computer Science, University of Stavanger, 4021 Stavanger, Norway; 3 Division of Medicine, Stavanger University Hospital, 4011 Stavanger, Norway; magnus.friestad.bjorkavoll-bergseth@sus.no; 4 Division of Cardiology, Stavanger University Hospital, 4011 Stavanger, Norway; orst@sus.no

**Keywords:** HRV, heart rate variability, artifact, correction, smartwatch, bicycle race, personal monitoring device

## Abstract

Heart rate variability (HRV) analysis can be a useful tool to detect underlying heart or even general health problems. Currently, such analysis is usually performed in controlled or semi-controlled conditions. Since many of the typical HRV measures are sensitive to data quality, manual artifact correction is common in literature, both as an exclusive method or in addition to various filters. With proliferation of Personal Monitoring Devices with continuous HRV analysis an opportunity opens for HRV analysis in a new setting. However, current artifact correction approaches have several limitations that hamper the analysis of real-life HRV data. To address this issue we propose an algorithm for automated artifact correction that has a minimal impact on HRV measures, but can handle more artifacts than existing solutions. We verify this algorithm based on two datasets. One collected during a recreational bicycle race and another one in a laboratory, both using a PMD in form of a GPS watch. Data include direct measurement of electrical myocardial signals using chest straps and direct measurements of power using a crank sensor (in case of race dataset), both paired with the watch. Early results suggest that the algorithm can correct more artifacts than existing solutions without a need for manual support or parameter tuning. At the same time, the error introduced to HRV measures for peak correction and shorter gaps is similar to the best existing solution (Kubios-inspired threshold-based cubic interpolation) and better than commonly used median filter. For longer gaps, cubic interpolation can in some cases result in lower error in HRV measures, but the shape of the curve it generates matches ground truth worse than our algorithm. It might suggest that further development of the proposed algorithm may also improve these results.

## 1. Introduction

Heart rate variability (HRV) analysis focuses on studying changes in intervals between heart beats. This analysis can be a useful tool to detect not only underlying heart issues, but also general health problems of both physical and psychological nature, including sports performance [1], cardiovascular events [2], stroke [3], and depression [4]. Presently, HRV parameters are used in exercise training to set optimal training loads and improve the performance of the person [5,6]. The most commonly used parameters are: low-frequency (LF) and high frequency (HF) modulation of R–R interval changes, standard deviation (SD) of all normal–normal (N–N) intervals (SDNN), the root mean square of SDs between adjacent N–N intervals (RMSSD), Standard Descriptors 1 and 2 (SD1 and SD2) representing the fast beat-to-beat variability in the R–R intervals, and longer-term variability respectively [7]. It was found that HRV parameters vary depending on the fitness level or physical condition in the given moment, e.g., stress level or illness [8,9]. In the case of training monitoring to maintain the sympathovagal balance, mainly frequency domain parameters, such as LF, HF and total power (TP), are significant [10]; in the case of age, influence on HRV SD1 parameter changes [11]; and athletic fitness is correlated with HF, SD1, SD2, and SDNN [12]. A frequent problem is the overtraining (OT) syndrome, which can be observed in changes of parameters from both time domain (RMSSDD and SDNN) and frequency domain (TP, LF, HF) [13,14].

Currently, heart rate data are usually acquired in controlled or semi-controlled conditions in laboratories. Since many of the typical HRV measures are sensitive to data quality, manual artifact correction is common in literature, both as an exclusive method or in addition to various filters. Manual methods include visual inspection and manually removing signal parts with artifacts [15,16,17,18], selection of artifact-free segments of recordings [19,20,21], or segments with error ration below 5% [22]. Some authors do not mention correction methods at all [23,24]. A frequent habit is also application of automatic methods followed by manual inspection to verify the correctness of the algorithms [25]; visually identified artifacts are corrected using appropriate filters [25] or manual intervention is needed by the software for artifact removal [26].

With proliferation of Personal Monitoring Devices with continuous beat-to-beat data collection, an opportunity opens for HRV analysis in a field setting. However, there are several challenges related to current artifact correction approaches, for example typical filters have a strong but often inconsistent influence on typical HRV measures. More advanced algorithms, available in commonly-used software packages, can usually avoid this problem, but leave some of the artifacts uncorrected. As a result, manual inspection remains necessary, but it is impractical or even infeasible with the vast amount of data from new devices.

To address these issues, we propose an algorithm for automated artifact correction that has a goal to minimize impact on HRV measures, but at the same time handles at least as many artifacts as existing solutions. We verify this algorithm based on two datasets. One collected during a recreational bicycle race and another one in a laboratory, both using a PMD in form of a GPS watch. Data include direct measurement of electrical myocardial signals using chest straps and direct measurements of power using a crank sensor (in case of race dataset), both paired with the watch. Both datasets provide HRV data that were collected continuously, in case of first dataset data are collected in a field setting and in case of the second one in a laboratory setting but using field-type equipment.

The reminder of this paper is organized as follows. In Section 3, we present problems encountered while analyzing HRV data collected in a field setting during a bicycle race. We propose an improved method for correction of artifact in HRV data in Section 4 and evaluate it in Section 5. We discuss the result, future work, and conclude in Section 6.

## 2. Existing Works

Heart Rate Variability signal may contain artifacts of biological or technical origin, in the case of both static and dynamic recordings [27,28]. Most common techniques of artifact correction in HRV include simple deleting of selected intervals [16,17,18], interpolation methods, such as linear or cubic spline [18,29,30,31], moving average windowing [32], and threshold filtering [32,33]. More advanced methods include comparison and merging [34], impulse rejection [35], nonlinear predictive interpolation [31], or the use of integral pulse frequency models [35]. Several studies use a combination of artifact correction methods [36,37]. There are several tools for HRV artifacts correction, such as ARTiiFACT software [38].

Giles and Draper [25] conducted in 2018 a comprehensive comparison of artifact correction methods for HRV during exercise. They classified six general types of artifacts ranging from minor discrepancies of 20 ms at a single interval, through single extra long or short interval, to multiple missing intervals. Artifacts were corrected by deletion and linear, cubic, or splice interpolation. Artifact correction implemented in Kubios HRV software was also evaluated. Kubios HRV [39] is a heart rate variability analysis software with built-in artifact detection and correction. It is commonly used for HRV analysis, in particular in medical and sports literature.

In their comparison study, Giles and Draper underlined that discarding sections with artifacts leads to bias in further analysis and even the smallest artifacts should be corrected to ensure correct calculation of HRV measures. At the same time, approaches to artifact correction vary greatly and sometimes are not even clearly specified in existing literature. The lack of standard practice in collection and processing of HRV data makes comparison between studies difficult and sometimes impossible.

To compare the approaches to artifact correction, the authors calculated a set of HRV measures, including SDNN (standard deviation of N–N intervals), RMSSD (root mean square differences of successive R–R intervals), SD1 and SD2 (standard deviations of Poincare scattergram), and low to high frequency ratio. Exercise intensity, in particular over 60% of VO2max, proved to have a significant impact on HRV measures. Linear interpolation often resulted in the lowest bias in HRV measures, but required manual detection of artifacts as other methods. Kubios was able to automatically detect most of artifacts, but did not reduce bias in HRV measures. Based on this study, no conclusion was reached regarding maximum number of artifacts that can be considered acceptable, but the authors suggested that numbers exceeding four should be treated with a particular caution.

In several studies, it has been confirmed that deletion technique gives best results in case of time domain analysis, however it influences significantly frequency domain parameters [40,41,42]. Interpolation methods of HRV artifact correction cause increase in LF and VLF components [42,43]. Research conducted by Soler et al. [40] suggests that the method having smallest influence n time and frequency domain parameters of HRV is nonlinear predictive interpolation (NPI).

Alcantara et al. [21] in 2020 evaluated impact of different levels of thresholds for artifact correction and how they impact quantification of HRV. Processing of HRV data was performed in Kubios. They discovered that choice of threshold level for applied filters can lead to clinically relevant differences in statistical analysis both in time and frequency domain. Moreover, optimal filter choice might also depend on the age of study subjects and intensity of the activity. Younger subjects require weaker filters in general, while higher intensity activities require stronger filters. As a result, filter choice becomes especially difficult in large studies with a variety of participants and dynamic (at will) activity level.

## 3. Example Problems

### 3.1. NEEDED Project

The basis for this work is a 2018 North Sea Race Endurance Exercise Study (NEEDED). It collected broad spectrum of data from 59 participants during a recreational 91-km long bike race. Mean age of participants was 50, and 46 participants were male. A CT scan was performed and showed early stages of atherosclerosis among 25 participants. All participants used the same continuous HRM (Garmin Forerunner 935, paired with Garmin HRM chest strap), and 40 of them had power meters (Stages^TM^). All data were downloaded in the raw format and processed using in-house software. This process, including details for handling typical data issues, was described by Wiktorski et al. [44]. The dataset is private.

**Figure 1:**
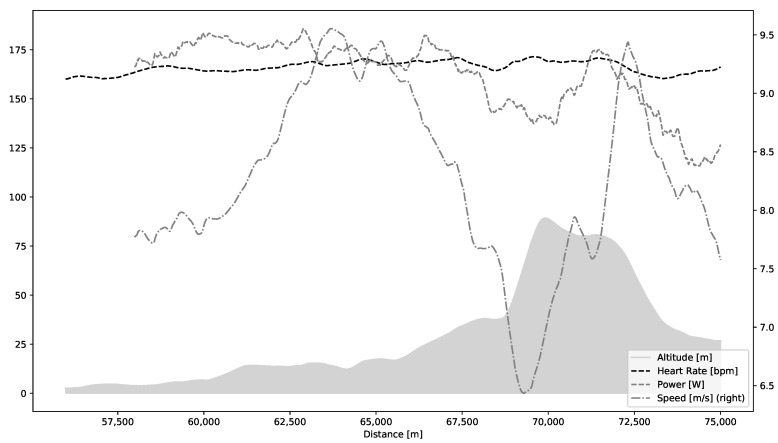
Altitude profile (gray-shaded area), heart rate, speed, and power for an example participant approaching and descending the hill, which is analyzed in the paper.

One of the unique aspects of this study is a possibility to use natural variations in the course of the race as a basis for an analysis of artifact correction at different work-loads and external conditions introducing mechanical noise. In the NEEDED study all participants follow the same course, but they choose their own pace. A steep hill after roughly two thirds of the course was selected for in-depth analysis; it is visualized in Figure 1. Due to covered distance, each participant experiences a fair level of exhaustion when approaching the hill. At the same time, the hill is steep enough to elicit maximal or close to maximal effort. It is followed by a short flat period and a descent. These provide additional information as participants perform differently depending on their strategies, health and fitness.

### 3.2. Typical Filter Setup

For the purpose of this work, we inspected HRV data of 23 participants of NEEDED project that had complete recordings of race data without major data quality problems. Such problems could include data missing for more than 10 s, unexplained stops, or inconsistent distance registration. Two specific issues were frequently present: sudden spikes in beat-to-beat interval duration (usually exceeding 300 ms over surrounding average level) and missing data points where empty values were recorded. These two issues are consistent with artifacts classification in existing literature. It is important to note that, while peaks and empty periods occurred in HRV recordings, the HR data remained continuous and also unaffected, as seen in Figure 1. There are two likely reasons for this discrepancy. Firstly, the HRM monitor automatically corrects HR data to produce a clean recording suitable for further analysis without preprocessing. Secondly, HRV data are stored separately from all other data (speed, distance, HR, etc.) in the FIT file (https://www.thisisant.com/developer/ant/licensing/flexible-and-interoperable-data-transfer-fit-protocol-license), which could lead to some unintended misalignment.

HRV analysis libraries, e.g., Python’s HRV (https://pypi.org/project/hrv/) or R’s RHRV (https://cran.r-project.org/web/packages/RHRV/vignettes/RHRV-quickstart.html), include a set of basic and advanced filters to address common problems in HRV data. Collection of HRV data during a sports event might naturally lead to more frequent and larger artifacts due to movement of the HR sensor. We found that it was usually necessary to apply two or more filters in combination and manually experiment with filter parameters to remove artifacts effectively.

**Figure 2:**
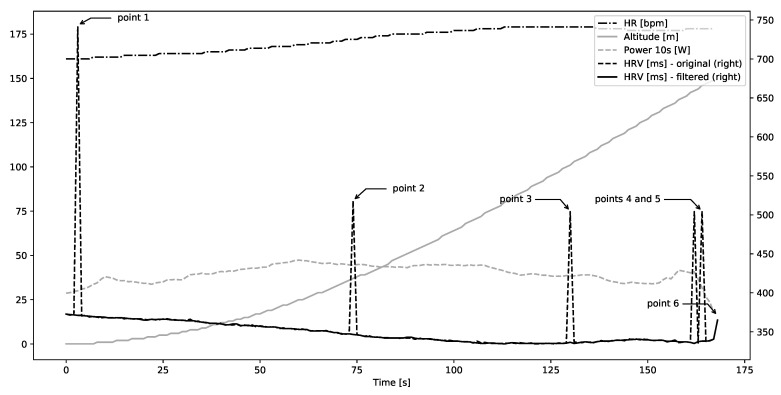
Example participant with single spaced-out artifacts and a double artifact in HRV beat-to-beat duration before filtering and after filtering.

In most cases, a combination of a median filter and Kubios-inspired threshold-based cubic interpolation was effective at removing the artifact while preserving, at least visually, the general shape of beat-to-beat interval curve. Single spaced-out artifacts (not exceeding 1 artifact per 50 s of recording) were removed by a combination of median filter with width 3 or 5. Such artifacts include Points 1, 2, and 3 in Figure 2. When multiple artifacts were clustered together, such as Points 4 and 5, a combination of median filter with width 5 (or in some cases 7) and Kubios-like interpolation with threshold 5 was necessary.

**Figure 3:**
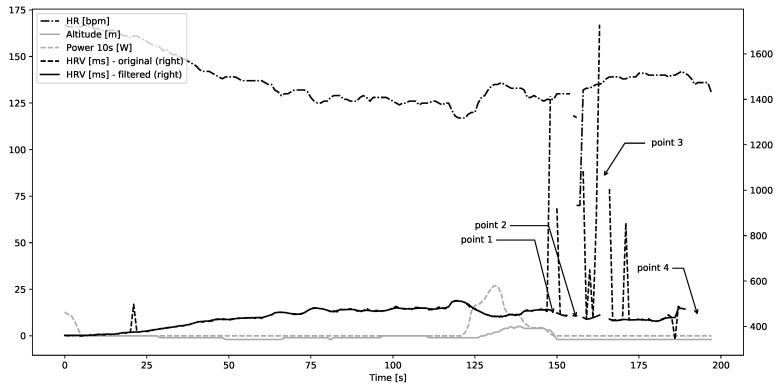
Example participant with multiple gaps in HRV beat-to-beat duration before filtering and after filtering.

However, such combination of filters was not enough to remove artifact towards beginning or end of the recording, such as Point 6 in that figure. Moreover, longer gaps, such as Points 1, 2, 3, and 4 in Figure 3, remained after filtering while peaks were removed. Such gaps should not be filled with simple interpolation due to the dynamics of heart rate.

In our experience and considering existing literature, it is currently necessary to perform individual inspection and manual experimentation with filter types and parameters. This fact becomes a key problem with growing amount of data coming from HRMs with continuous beat-to-beat data recording and in an end-user scenario where an analytic algorithm is to be implemented on a smart watch or in a smart phone application.

### 3.3. Filters’ Impact on HRV Measures

An additional, sometimes overlooked, issue is that application of filters usually has a noticeable but also occasionally unpredictable impact on HRV measures. It might be particularly visible when filters are applied as a blanket solution in cases when manual inspection is impractical or infeasible.

To demonstrate this, we chose a set of participants without visible artifacts; applied typical filters to a short period of HRV data; and then compared typical HRV measures before and after filtering. This way the ground truth is known and can be used to measure changes filters introduce. Should we choose data with pre-existing artifacts, we would not have the necessary information to perform such an evaluation.

Based on the inspection of data from hill ascent and descent we observed that the ascent part is prone to many artifacts. It is not surprising since it is one of the most strenuous periods of the race. During the rest on the top of the hill, there were the fewest artifacts, with many participants without any. However, it is not representative of a typical active exercise condition. The descent part still exhibits many artifacts, but it was possible to identify several participants without artifacts. At the same time, this part can be considered representative of an active exercise in a sense that an activity is present and is not fully predictable.

In Table 1, Table 2 and Table 3, we present values for RMSSD (root-mean square differences of successive R–R intervals), SDNN (standard deviation of N–N intervals), SDSD (standard deviation of successive R–R interval differences), Total Power (over all frequency bands), LF (low-frequency modulation of R–R interval changes), HF (high frequency modulation of R–R interval changes) and SD1 and SD2 (standard deviations of Poincare scattergram). These cover a mix of time domain, frequency domain, and non-linear analysis of HRV.

Usually, RMSSD, SDSD, and SD1 are reduced by a factor of 3 by the time median filter of width 7 was applied. This factor increases approximately linearly with growing width of the filter. However, for some participants who otherwise seem similar in terms of values of measures, population data, etc., we see reduction just by 25%. A more advanced Kubios-inspired threshold filter with cubic interpolation does not cause such changes, but it cannot rectify many artifacts. Many gaps and sudden peaks due to missing beats will remain; and in particular longer holes are not filled at all.

It means that adding a filter might have an impact on interpretation of HRV information even when applying the same filter consistently to potentially similar participants. This problem is not new and a need for more research and standardization in filter application is mentioned in the review works discussed in Section 1.

## 4. Proposed Method

In this section, we introduce two algorithms that address preservation of HRV measures during typical HRV artifact correction procedures—peak correction and gap filling.

In Algorithm 1, we present an approach for correcting single peaks in HRV. In principle, the approach can be applied to peaks of any size; however, available literature suggests that peaks exceeding local average beat-to-beat duration by a factor of 3 or 4 should be treated with additional care. The information on how the average value is procured is often omitted in publications or not given with enough detail to allow replication. Usually, it is calculated by either: (1) global mean or median for very short HRV samples; or (2) local sliding-window mean or median filtering for longer samples. The window length is a user-specified parameter provided as an input to the filtering process.

Input to the algorithms consists of HRV data in the form of list of beat-to-beat durations of length *N*; values are usually recorded in milliseconds. The result is a corrected list of beat-to-beat durations, of length *N+*, which will be longer than the input list. The actual change in the length of the list will depend on the amount and size of detected peaks, a value which is reflected in the variable *total_new*.

On Line 1, existing beat-to-beat durations are transferred from input to output variable, as for now without any corrections. On Line 2, we detect peaks, obtaining a list of tuples with location of each peak and its estimated magnitude with respect to surrounding data. Description of the peak detection algorithm *detect_peaks* is omitted as it can be implemented simply as a threshold-based or value discontinuity-based identification of local extrema. Such algorithms are available in common data analytic libraries, for example in SciPy (https://docs.scipy.org/doc/scipy/reference/generated/scipy.signal.argrelextrema.html).

On Line 3, *total_new* variable is reset; it will hold a total number of added beats when correcting the detected peaks. On Line 4, we start iterating over each detected peak. We check first if the height of the peak *est_beats* does not exceed surrounding values by more than 3 on Line 5, and, if it does, the area is changed into a gap on Line 13 to be filled by a more advanced algorithm. Total number of beats added is updated on Line 14.
**Algorithm 1:** Algorithm for correcting short peaks in beat-to-beat Heart Rate Variability data
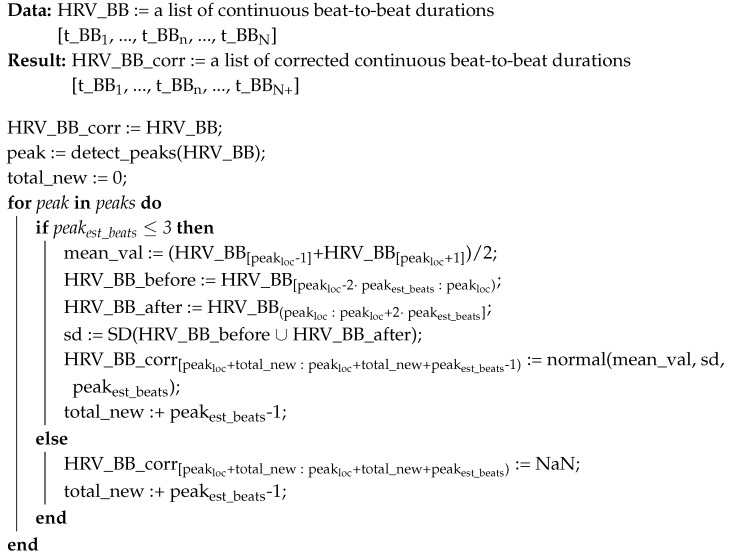


Further, on Line 6, expected value of the beat-to-beat duration is estimated based on the surrounding area. *Loc* parameter refers to location of the peak in the list *HRV_BB*. On Lines 7–9, values of estimated standard deviation are calculated based on periods before and after the detected peak, taking into account periods of time equal to 2 multiples of estimated peak height. Finally, beat-to-beat durations are generated using a normal distribution. The amount of generated values is based on the output of peak detection algorithm—effectively an integer multiple of average of surrounding beat-to-beat durations—and mean value and standard deviation as calculated on Lines 6–9. It is important to notice that on Line 10 indices in the result variable *HRV_BB_corr* are adjusted for the changing length with each detected peak; the variable *total_new* that maintains the correction sum is updated on Line 11.

In Algorithm 2, we present an approach to filling gaps that occur either naturally or as a byproduct of removing larger peaks—as in Algorithm 1. Input to the algorithm consists of HRV data in the form of list of beat-to-beat durations, usually in milliseconds, and HR data in the form of a time-ordered list of equisampled, instantaneous pulse values, usually in beats per minute. Such HR data are recorded independently of HRV by major continuous HRMs. An important observation is that sum of all the values of *HRV_BB* is equal to the total time for which HR is recorded, as represented in Equation (1). In principle, these two values should be identical, but, in practice, small misalignments might occur. These misalignments do not accumulate with time so any error will be minor and independent of the length of input. The result of the algorithm is a corrected list of beat-to-beat durations, which in this case is of the same length as the input.

(1) $$\sum_{}^{N}HRV\_BB≅T$$

**Algorithm 2:** Algorithm for correcting missing values in beat-to-beat Heart Rate Variability data

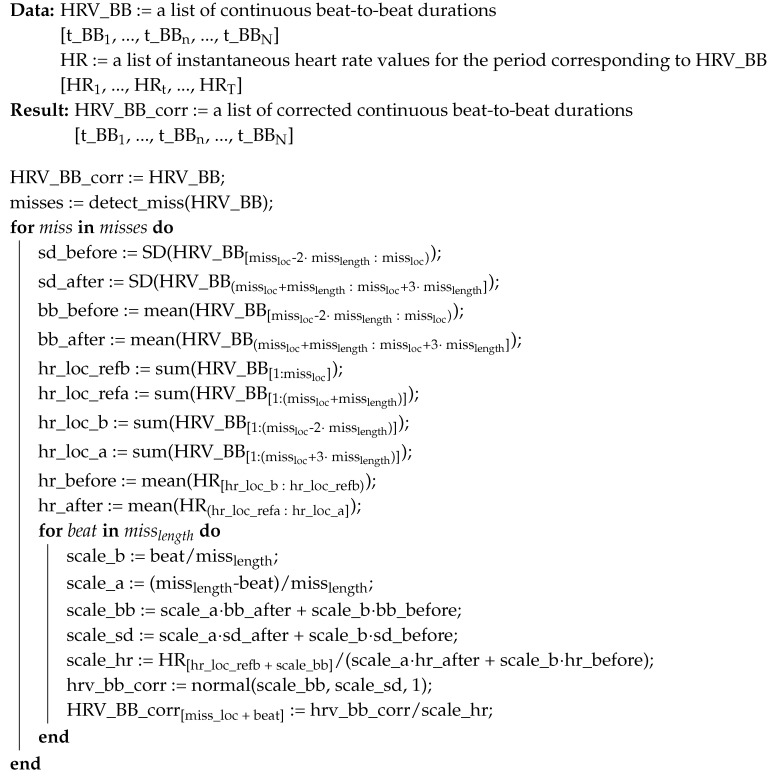



On Line 1, existing beat-to-beat durations are transferred from input to output, as for now without any corrections. On Line 2, we detect gaps, obtaining a list of tuples with location of each gap and its width. We choose to omit a description of the gap detection algorithm *detect_miss* as it effectively amounts to identification of non-number values in the *HRV_BB* list. Such detection is usually a built-in feature of most data analysis frameworks such as *Pandas* or *R*.

On Line 4, we start iterating over each gap, and, on Lines 4–7, standard deviation and beat-to-beat durations are calculated for periods before and after the gap. A time period double that of the gap size is taken into account on each side. On Lines 8–13, reference points for locating relevant periods in HR list are calculated, translating indices between HRV beat-to-beat durations to HR time recordings based on the dependence from Equation (1).

Having determined the reference points, missing beat-to-beat distances can be on Lines 14–22. For each beat, we calculate how far it is from the beginning and the end of the gap, on Lines 15 and 16, respectively. Then, estimated beat-to-beat duration and standard deviation are calculated using basic interpolation on Lines 17 and 18. The estimated beat-to-beat duration allows us to identify an estimated new location in the HR input list and retrieve the respective value.

This HR value is then used, on Line 19, to calculate a corrective scale factor that will adjust estimated beat-to-beat duration for any non-Linear changes to heart rate. Heart rate slowing down or accelerating is an import factor that influences HRV beat-to-beat durations in particular over longer gaps. Should the heart rate simply change linearly between beginning and end of the gap, this factor will be equal to one. On Line 20, a preliminary beat-to-beat duration value is generated using normal distribution based on the interpolated values, and, on Line 21, it is adjusted with the corrective scale factor.

## 5. Results

We implemented Algorithms 1 and 2 presented in the previous section in Python using Pandas and SciPy, and then performed proof-of-concept tests on the examples presented in Section 3. For this purpose, we also extended the cubic spline interpolation implementation. We demonstrated earlier that the existing implementation did not manage to fill in longer gaps, despite no such limitation should theoretically exist. It is necessary to underline that the presented results are an indication of the potential usefulness of this concept, rather than an ultimate proof. The main reason is the relatively small sample used for testing and shortage of standardized open datasets; we discuss this problem further in Section 6.

**Figure 4:**
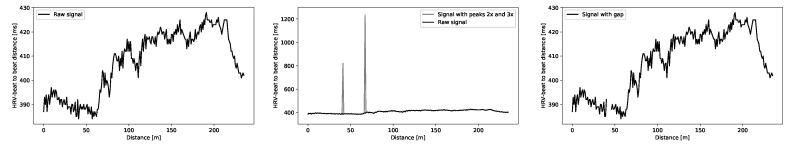
Example HRV signals used in algorithms’ validation: raw HRV signal; signal with manually introduced peak artifacts; and signal with manually introduced gap artifact.

The algorithms were tested on real data. To verify the performance of the proposed algorithms, five types of artifacts were manually introduced to raw HRV signals (Figure 4): (1) peak of the size of double mean value of the signal and width of a single sample; (2) peak of size of triple mean value of the signal and width of a single sample; and (3) gaps of size 3, 5. and 7 as undefined samples in the signal. These artifacts were introduced in random parts of the raw signal. Parameters of HRV signal from time and frequency domain were calculated for the raw signals and compared with the parameters calculated for signals initially contaminated with manually introduced artifacts and filtered using reference and proposed methods.

Data were acquired from two databases: recordings from NEEDED project and signals from PhysioNet simultaneous physiological measurements [45,46]. From NEEDED project, HRV signal and Heart Rate signal, recorded using Garmin Forerunner 935, paired with Garmin HRM chest strap, were used in the testing process. From PhysioNet database, HRV signal and Heart Rate signal, recorded using Polar RS800 Multi paired with chest strap with flat electrodes made of conductive rubber, were used in the testing process. The part of the signal related to 5 min uphill walking on the treadmill (15% track inclination, 1.2 m/s) was extracted for processing by Algorithms 1 and 2. Algorithm 1 was applied to eight recordings from NEEDED database that did not contain spike artifacts in the original signal and to 13 signals from PhysioNet database. In Table 4, Table 5 and Table 6, we present results of correction of randomly introduced single peaks for NEEDED database. Results for PhysioNet database are given in Table 7 and Table 8. Peaks that have an approximate size two and three times the surrounding average value are considered. We compare our approach from Algorithm 1 with the earlier used median filter with width 3 and Kubios-like interpolation with threshold value 5. Table 9 and Table 10 present summary of mean error calculated for the selected HRV parameters for all analyzed signals. Our approach provides good results with error at level of 2%, much better than the median filter (16% on average) and on par with the interpolation approach. However, the interpolation approach is not able to remove larger peaks (with amplitude four or five times the surrounding average value) or series of smaller ones. In contrast, our approach would also work for larger spikes, of values above three times the mean of the signal if necessary. Since our method is nondeterministic, all experiments were repeated 50 times and average values are presented in Table 4, Table 5, Table 6, Table 7, Table 8, Table 9, Table 10, Table 11, Table 12 and Table 13.

**Figure 5:**
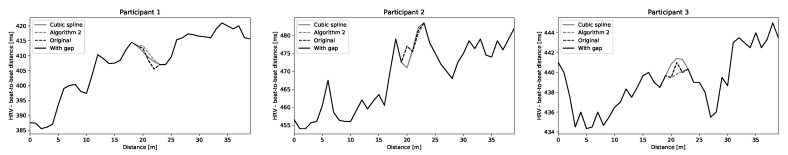
Comparing shape of the signal reconstructed by cubic spline interpolation and the proposed algorithm for gap size 3 for all three participants (NEEDED database).

**Figure 6:**
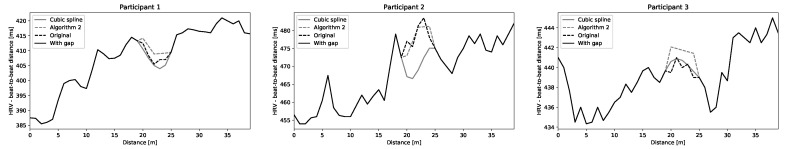
Comparing shape of the signal reconstructed by cubic spline interpolation and the proposed algorithm for gap size 5 for all three participants (NEEDED database).

**Figure 7:**
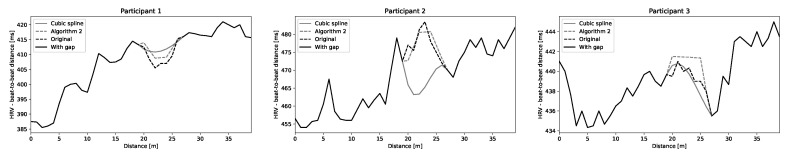
Comparing shape of the signal reconstructed by cubic spline interpolation and the proposed algorithm for gap size 7 for all three participants (NEEDED database).

**Figure 8:**
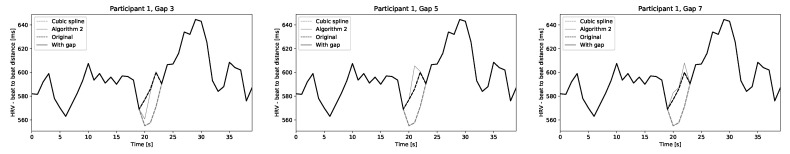
Comparing shape of the signal reconstructed by cubic spline interpolation and the proposed algorithm for Participant 1 for gap sizes 3, 5 and 7 (PhysioNet database).

**Figure 9:**
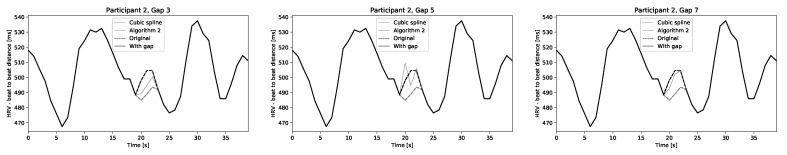
Comparing shape of the signal reconstructed by cubic spline interpolation and the proposed algorithm for Participant 2 for gap sizes 3, 5 and 7 (PhysioNet database).

**Figure A9:**
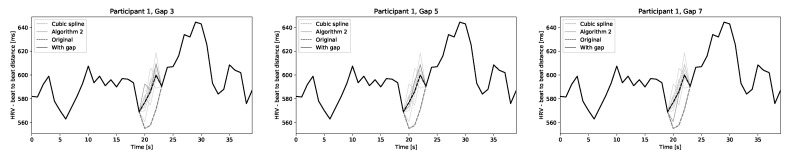
Participant 1 with multiple gaps in HRV beat-to-beat duration before filtering and after filtering (PhysioNet database).

**Figure A10:**
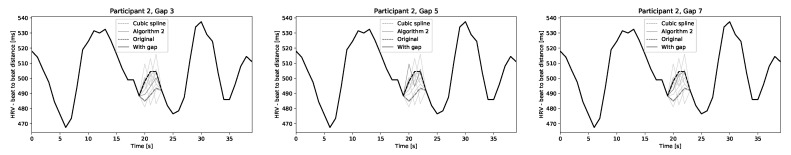
Participant 2 with multiple gaps in HRV beat-to-beat duration before filtering and after filtering (PhysioNet database).

**Figure A11:**
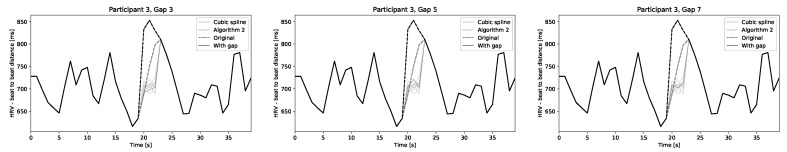
Participant 3 with multiple gaps in HRV beat-to-beat duration before filtering and after filtering (PhysioNet database).

**Figure A12:**
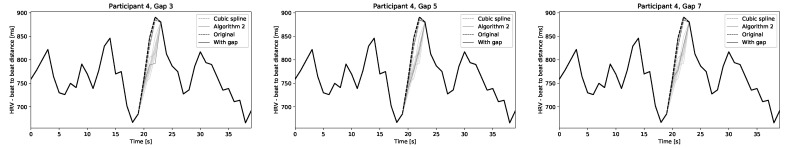
Participant 4 with multiple gaps in HRV beat-to-beat duration before filtering and after filtering (PhysioNet database).

**Figure A13:**
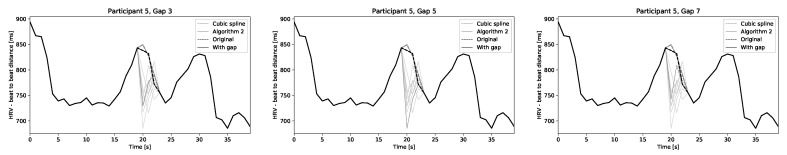
Participant 5 with multiple gaps in HRV beat-to-beat duration before filtering and after filtering (PhysioNet database).

**Figure A14:**
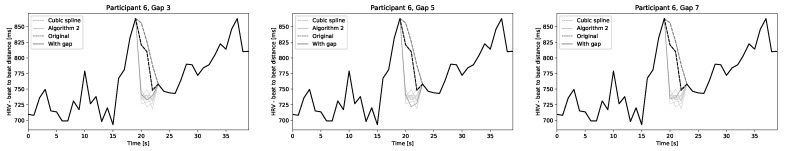
Participant 6 with multiple gaps in HRV beat-to-beat duration before filtering and after filtering (PhysioNet database).

**Figure A15:**
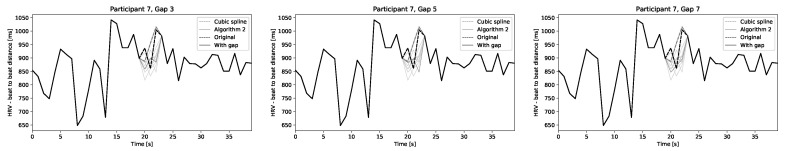
Participant 7 with multiple gaps in HRV beat-to-beat duration before filtering and after filtering (PhysioNet database).

**Figure A16:**
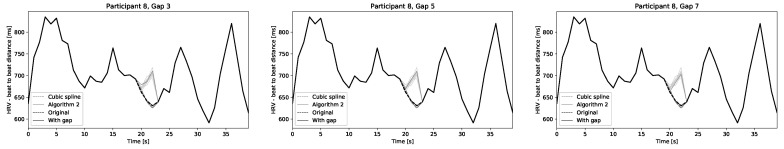
Participant 8 with multiple gaps in HRV beat-to-beat duration before filtering and after filtering (PhysioNet database).

**Figure A17:**
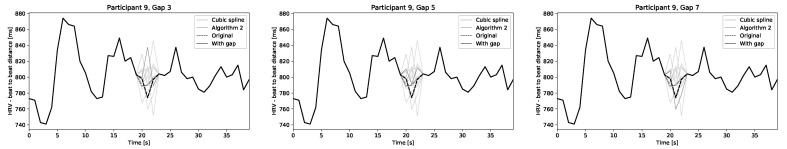
Participant 9 with multiple gaps in HRV beat-to-beat duration before filtering and after filtering (PhysioNet database).

**Figure A18:**
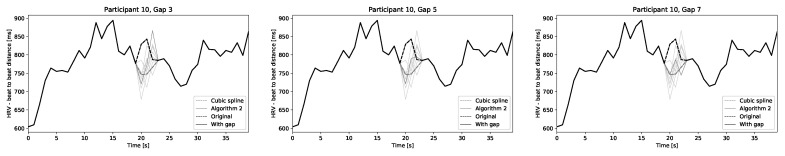
Participant 10 with multiple gaps in HRV beat-to-beat duration before filtering and after filtering (PhysioNet database).

**Figure A19:**
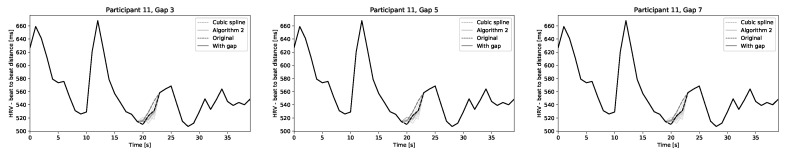
Participant 11 with multiple gaps in HRV beat-to-beat duration before filtering and after filtering (PhysioNet database).

**Figure A20:**
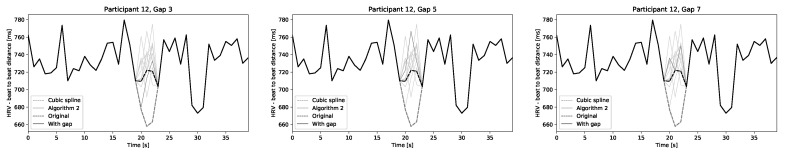
Participant 12 with multiple gaps in HRV beat-to-beat duration before filtering and after filtering (PhysioNet database).

**Figure A21:**
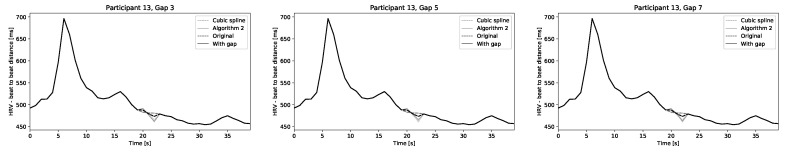
Participant 13 with multiple gaps in HRV beat-to-beat duration before filtering and after filtering (PhysioNet database).

Table 11, Table 12 and Table 13 present results of filling of randomly introduced gaps of size 3, 5, and 7 beats for three participants from NEEDED project. Table 14 and Table 15 show values calculated for two signals from PhysioNet database. The results for Algorithm 2 are mean values from 50 iterations of the algorithm. Standard deviation values ranged from $2\times 10^{-7}\;\mathrm{to}\;1.3\times 10^{-1}$ for NEEDED database. In the case of PhysioNet data, the SD values vary from 0.003 for time-domain and nonlinear parameters up to 11.51 for frequency-domain parameters as they reach values above 2000. We compare our approach with cubic spline interpolation (which is commonly used for such purpose, e.g., by Kubios). The values of mean error are presented in Table 16 and Table 17. The results are fairly similar. Our method usually introduces a smaller error for small gaps, but can introduce larger differences for longer gaps, especially for frequency-based HRV measures. However, if we inspect the generated data visually, the shape of the HRV beat-to-beat durations reconstructed using the proposed algorithm is more similar to the original than in the case of spline interpolation. Several examples are presented in Figure 5 for a gap of three beats, in Figure 6 for a gap of five beats, and in Figure 7 for a gap of seven beats for NEEDED project and Figure 8 and Figure 9 for PhysioNet database. The plots for 23 participants of the NEEDED project are presented in Appendix A and plots generated for signals from PhysioNet database are presented in Appendix B. For all signals from NEEDED project, the curves generated for filling the gaps by Algorithm 2 overlapped as the SD of the signal was very low, at the level of 0.03. In the case of signals from PhysioNet database, there are significant fluctuations visible in Figure A9, Figure A10, Figure A11, Figure A12, Figure A13, Figure A14, Figure A15, Figure A16, Figure A17, Figure A18, Figure A19, Figure A20 and Figure A21, which is caused by much larger values of SD for these signals, at the level of 20. We can notice that sometimes there is a small shift in time in the data generated by the proposed approach, which might be a source of increased error.

## 6. Discussion and Conclusions

Many HRV measures are sensitive to data quality and filtering is usually applied to combat the problem. At the same time, there is a consensus in existing literature that HRV measures are influenced by applied filtering to an extent to that produced clinically relevant differences in data interpretation. There is also lack of standardization and openness about types of filters applied and parameters used.

The existing manual approaches to filter selection and artifact correction in HRV data are not sustainable with proliferation of Personal Monitoring Devices capable of continuous recording of beat-to-beat HRV data. To address these problems, we propose two algorithms for automated artifact correction that were designed to have a minimal impact on HRV measures and to handle more artifacts than existing solutions.

In this paper, we present the algorithms and the studies to validate their initial versions using datasets. One collected during a recreational bicycle race and another one in a laboratory setting. Our findings suggest that the proposed algorithm can correct more artifacts than existing methods without a need for manual support or parameter tuning. At the same time, the error introduced to HRV measures for peak correction and shorter gaps is similar to the best existing solution (Kubios-inspired threshold-based cubic interpolation) and better than commonly used median filter. For longer gaps, cubic interpolation can in some cases result in lower error in HRV measures. At the same time, in this case, we observe that the shape of the curve reconstructed by our algorithm matches ground truth better than cubic interpolation. It might suggest that further development of the algorithm may also improve these results.

One of the challenges in HRV research is a shortage of standardized and open datasets. Most of the open HRV datasets are short in duration, collected during rest, and using ECG equipment. A search for heart rate variability data in PhysioNet [46] reveals only five results, of which after further inspection only two are to some extent relevant. The first one contains a computer model to generate data. The second one [45] contains real data collected with a consumer-level device and became available only during the review stages of this paper. Validation based on this dataset is included and consistent with earlier results. The problem of lack of open datasets and general standardization has been noticed by other authors, as mentioned in Section 2.

We plan further development of algorithm to improve results in filling longer gaps. The current version of the algorithm uses beat-to-beat distances and heart rate as inputs. Newer PMDs are now frequently capable of recording breath rate, which has an impact on HRV. We plan to investigate how these data could be used to improve accuracy of the algorithm; they might have a particular impact for long gaps. We also plan an extended verification by identifying new relevant subsections of NEEDED dataset and more participants from the new dataset made available recently on PhysioNet.

## Figures and Tables

**Table 1:** Selected typical HRV measures for a healthy Participant 1 during hill descent.

Median Len	Threshold Len	RMSSD	SDNN	SDSD	Total Power	LF	HF	SD1	SD2
-	-	3.09	13.13	3.10	49.52	5.42	2.21	2.19	18.44
3	0	1.61	13.09	1.61	49.65	4.79	2.08	1.14	18.40
0	3	3.09	13.13	3.10	49.53	5.42	2.21	2.19	18.44
3	3	1.61	13.09	1.61	49.65	4.80	2.08	1.14	18.48
0	5	3.09	13.13	3.10	49.53	5.42	2.21	2.19	18.44
5	0	1.39	13.01	1.39	48.66	4.63	1.56	0.98	18.38
5	5	1.39	13.01	1.39	48.66	4.63	1.56	0.98	18.38
7	5	1.12	12.95	1.12	47.37	4.37	0.83	0.80	18.30

**Table 2:** Selected typical HRV measures for a healthy Participant 2 during hill descent.

Median Len	Threshold Len	RMSSD	SDNN	SDSD	Total Power	LF	HF	SD1	SD2
-	-	5.92	13.78	5.96	63.59	12.32	6.98	4.22	19.02
3	0	2.64	13.45	2.64	59.65	9.82	5.42	1.87	18.93
0	3	5.95	13.78	5.96	63.59	12.32	6.98	4.22	19.02
3	3	2.64	13.45	2.64	59.65	9.82	5.42	1.87	18.93
0	5	5.95	13.78	5.96	63.59	12.32	6.98	4.22	19.02
5	0	2.13	13.38	2.13	58.64	10.22	4.30	1.50	18.86
5	5	2.13	13.38	2.13	58.64	10.27	4.30	1.50	18.86
7	5	1.71	13.32	1.71	55.06	8.29	2.04	1.21	18.80

**Table 3:** Selected typical HRV measures for a healthy Participant 3 during hill descent.

Median Len	Threshold Len	RMSSD	SDNN	SDSD	Total Power	LF	HF	SD1	SD2
-	-	4.24	22.26	4.22	225.54	13.45	1.95	2.98	31.34
3	0	3.49	22.02	3.46	221.81	12.88	1.51	2.45	31.04
0	3	4.24	22.26	4.22	225.54	13.45	1.95	2.98	31.34
3	3	3.49	22.02	3.46	221.81	12.88	1.51	2.45	31.04
0	5	4.24	22.26	4.22	225.54	13.45	1.95	2.98	31.34
5	0	3.26	21.86	3.23	217.11	12.24	1.15	2.28	30.84
5	5	3.26	21.86	3.23	217.11	12.24	1.16	2.28	30.84
7	5	3.01	21.94	2.97	208.87	10.92	0.61	2.10	30.31

**Table 4:** Typical HRV measures after applying median and threshold filters, and the proposed algorithms for various peak sizes, compared to the HRV measures in the original signal for Participant 1 (NEEDED database).

Signal	Method	RMSSD	SDNN	SDSD	Total Power	LF	HF	SD1	SD2	Mean Error
Original		3.09	13.13	3.10	49.52	5.43	2.21	2.19	18.44	
Peak 2x	Median w = 3	1.67	13.01	1.67	49.07	4.91	2.26	1.18	18.36	19.0%
	Thr m = 5	3.05	13.09	3.05	49.30	5.46	2.31	2.15	18.39	1.3%
	Algorithm 1 (StDev.)	3.07 (0.02)	13.16 (0.02)	3.07 (0.02)	49.58 (0.14)	5.40 (0.03)	2.24 (0.02)	2.17 (0.01)	18.49 (0.03)	0.6%
Peak 3x	Median w = 3	1.67	13.01	1.67	49.03	4.95	2.40	1.18	18.36	19.7%
	Thr m = 5	3.05	13.10	3.05	49.34	5.43	2.36	2.15	18.39	1.5%
	Algorithm 1 (StDev.)	3.07 (0.02)	13.16 (0.02)	3.07 (0.02)	49.58 (0.14)	5.40 (0.03)	2.24 (0.02)	2.17 (0.01)	18.49 (0.03)	0.7%

**Table 5:** Typical HRV measures after applying median and threshold filters, and the proposed algorithms for various peak sizes, compared to the HRV measures in the original signal for Participant 2 (NEEDED database).

Signal	Method	RMSSD	SDNN	SDSD	Total Power	LF	HF	SD1	SD2	Mean Error
Original		5.94	13.77	5.96	63.58	12.32	6.98	4.21	19.02	
Peak 2x	Median w = 3	2.63	13.44	2.63	58.58	9.64	5.47	1.86	18.92	27.6%
	Thr t = 5	5.94	13.79	5.95	62.62	12.04	6.98	4.21	19.05	0.6%
	Algorithm 1 (StDev.)	5.97 (0.04)	13.85 (0.03)	5.98 (0.04)	62.98 (0.35)	12.09 (0.02)	6.99 (0.04)	4.23 (0.03)	19.12 (0.05)	0.6%
Peak 3x	Median w = 3	2.63	13.45	2.63	57.51	9.45	5.59	1.86	18.92	27.8%
	Thr m = 5	5.94	13.80	5.96	61.57	11.74	7.06	4.21	19.06	1.2%
	Algorithm 1 (StDev.)	5.97 (0.04)	13.84 (0.03)	5.98 (0.04)	62.90 (0.36)	12.08 (0.02)	6.99 (0.03)	4.23 (0.03)	19.11 (0.03)	0.7%

**Table 6:** Typical HRV measures after applying median and threshold filters, and the proposed algorithms for various peak sizes, compared to the HRV measures in the original signal for Participant 3 (NEEDED database).

Signal	Method	RMSSD	SDNN	SDSD	Total Power	LF	HF	SD1	SD2	Mean Error
Original		4.24	22.26	4.22	225.53	13.46	1.95	2.98	31.34	
Peak 2x	Median w = 3	3.49	22.04	3.46	227.83	12.13	1.78	2.45	31.07	9.3%
	Thr m = 5	4.24	22.24	4.22	227.00	12.62	1.84	2.98	31.31	1.6%
	Algorithm 1 (StDev.)	4.47 (0.25)	22.29 (0.04)	4.45 (0.25)	228.94 (2.90)	12.54 (0.12)	2.10 (0.31)	3.15 (0.18)	31.37 (0.06)	4.1%
Peak 3x	Median w = 3	3.50	22.04	3.46	232.51	11.38	1.85	2.45	31.08	9.8%
	Thr m = 5	4.24	22.24	4.22	229.19	11.91	1.84	2.98	31.31	2.4%
	Algorithm 1 (StDev.)	4.42 (0.19)	22.03 (0.06)	4.40 (0.19)	229.54 (3.52)	12.54 (0.16)	2.20 (0.50)	3.11 (0.14)	31.38 (0.07)	4.3%

**Table 7:** Typical HRV measures after applying median and threshold filters, and the proposed algorithms for various peak sizes, compared to the HRV measures in the original signal for Participant 1 (PhysioNet database).

Signal	Method	RMSSD	SDNN	SDSD	Total Power	LF	HF	SD1	SD2	Mean Error
Original		0.35	2.69	0.35	1.52	0.09	0.50	0.25	3.80	
Peak 2x	Median w = 3	0.29	2.68	0.29	1.46	0.09	0.47	0.21	3.79	8.7%
	Thr m = 5	0.35	2.70	0.35	1.47	0.09	0.48	0.25	3.80	1.7%
	Algorithm 1 (StDev.)	0.35 (3.4 × 10${}^{-3}$)	2.69 (9.3 × 10${}^{-4}$)	0.35 (3.4 × 10${}^{-3}$)	1.49 (7.5 × 10${}^{-3}$)	0.09 (2.1 × 10${}^{-3}$)	0.49 (3.2 × 10${}^{-3}$)	0.25 (2.4 × 10${}^{-3}$)	3.80 (1.3 × 10${}^{-3}$)	1.3%
Peak 3x	Median w = 3	0.29	2.68	0.29	1.45	0.10	0.46	0.21	3.79	9.5%
	Thr m = 5	0.35	2.70	0.35	1.47	0.09	0.48	0.25	3.80	1.7%
	Algorithm 1 (StDev.)	0.35 (5.5 × 10${}^{-3}$)	2.69 (7.9 × 10${}^{-4}$)	0.35 (5.5 × 10${}^{-3}$)	1.49 (6.2 × 10${}^{-3}$)	0.09 (1.7 × 10${}^{-3}$)	0.49 (2.6 × 10${}^{-3}$)	0.25 (3.9 × 10${}^{-3}$)	3.80 (1.1 × 10${}^{-3}$)	1.2%

**Table 8:** Typical HRV measures after applying median and threshold filters, and the proposed algorithms for various peak sizes, compared to the HRV measures in the original signal for Participant 2 (PhysioNet database).

Signal	Method	RMSSD	SDNN	SDSD	Total Power	LF	HF	SD1	SD2	Mean Error
Original		0.17	2.85	0.16	1.36	0.11	0.07	0.11	4.02	
Peak 2x	Median w = 3	0.14	2.85	0.14	1.35	0.11	0.06	0.10	4.02	6.6%
	Thr m = 3	0.17	2.85	0.16	1.31	0.10	0.06	0.11	4.02	3.0%
	Algorithm 1 (StDev.)	0.17 (1.3 × 10${}^{-2}$)	2.84 (5.2 × 10${}^{-4}$)	0.17 (1.3 × 10${}^{-2}$)	1.32 (5.4 × 10${}^{-3}$)	0.11 (3.2 × 10${}^{-3}$)	0.07 (1.9 × 10${}^{-3}$)	0.12 (9.5 × 10${}^{-3}$)	4.01 (5.7 × 10${}^{-4}$)	2.5%
Peak 3x	Median w = 3	0.14	2.85	0.14	1.30	0.10	0.06	0.10	4.02	8.5%
	Thr m = 3	0.17	2.85	0.16	1.31	0.10	0.06	0.11	4.02	3.0%
	Algorithm 1 (StDev.)	0.18 (8.5 × 10${}^{-3}$)	2.84 (3.0 × 10${}^{-4}$)	0.17 (8.7 × 10${}^{-3}$)	1.32 (3.9 × 10${}^{-3}$)	0.11 (2.3 × 10${}^{-3}$)	0.06 (1.4 × 10${}^{-3}$)	0.12 (6.2 × 10${}^{-3}$)	4.01 (3.2 × 10${}^{-4}$)	2.5%

**Table 9:** Mean error for typical HRV measures calculated after applying median and threshold filters, and the proposed algorithms for various peak sizes (NEEDED database).

Signal	Method	RMSSD	SDNN	SDSD	Total Power	LF	HF	SD1	SD2
Peak 2x	Median w = 3	52.52%	2.29%	52.61%	6.11%	31.76%	23.67%	52.61%	0.71%
	Thr m = 3	0.35%	0.12%	0.35%	0.79%	2.00%	3.99%	0.35%	0.12%
	Algorithm 1	1.37%	0.24%	1.39%	0.71%	2.15%	2.18%	1.31%	0.25%
Peak 3x	Median w = 3	52.52%	2.29%	52.61%	6.56%	33.53%	24.56%	52.61%	0.71%
	Thr m = 3	0.43%	0.13%	0.43%	1.58%	4.23%	7.98%	0.43%	0.14%
	Algorithm 1	1.28%	0.25%	1.30%	0.78%	2.18%	2.74%	1.30%	0.25%

**Table 10:** Mean error for typical HRV measures calculated after applying median and threshold filters, and the proposed algorithms for various peak sizes (PhysioNet database).

Signal	Method	RMSSD	SDNN	SDSD	Total Power	LF	HF	SD1	SD2
Peak 2x	Median w = 3	5.38%	0.17%	5.67%	1.64%	3.32%	4.80%	5.67%	0.16%
	Thr m = 3	0.12%	0.01%	0.12%	2.61%	6.14%	6.50%	0.12%	0.01%
	Algorithm 1	2.17%	0.37%	2.33%	2.06%	2.39%	3.17%	2.40%	0.39%
Peak 3x	Median w = 3	5.38%	0.17%	5.67%	2.78%	5.77%	8.47%	5.67%	0.16%
	Thr m = 3	0.12%	0.01%	0.12%	2.61%	6.14%	6.50%	0.12%	0.01%
	Algorithm 1	2.31%	0.39%	2.46%	1.92%	3.41%	4.12%	2.49%	0.41%

**Table 11:** Typical HRV measures after applying cubic spline interpolation and the proposed algorithms for various gap sizes, compared to the HRV measures in the original signal for Participant 1 (NEEDED database).

Signal	Method	RMSSD	SDNN	SDSD	Total Power	LF	HF	SD1	SD2	Mean Error
Original		2.32	12.95	2.39	45.96	6.67	4.91	1.64	18.25	
Gap w = 3	Cubic spline	2.28	12.95	2.29	45.38	6.59	4.38	1.61	18.24	2.4%
	Algorithm 2	2.29	12.96	2.30	45.56	6.60	4.56	1.62	18.26	1.7%
	(StDev.)	(153 × 10${}^{-5}$)	(6.73 × 10${}^{-6}$)	(1.55 × 10${}^{-5}$)	(2.88 × 10${}^{-4}$)	(3.27 × 10${}^{-5}$)	(2.83 × 10${}^{-4}$)	1.09 × 10${}^{-5}$)	(9.03 × 10${}^{-6}$)
Gap w = 5	Cubic spline	2.34	12.96	2.35	47.32	7.00	6.05	1.66	18.25	4.2%
	Algorithm 2	2.27	12.96	2.28	44.83	6.53	3.75	1.61	18.26	4.3%
	(StDev.)	(2.60 × 10${}^{-5}$)	(6.24 × 10${}^{-6}$)	(2.62 × 10${}^{-5}$)	(4.50 × 10${}^{-4}$)	(5.63 × 10${}^{-5}$)	(4.83 × 10${}^{-4}$)	(1.85 × 10${}^{-5}$)	(8.32 × 10${}^{-6}$)
Gap w = 7	Cubic spline	2.18	12.96	2.19	43.69	6.42	2.36	1.55	18.27	9.9%
	Algorithm 2	2.24	12.97	2.24	44.69	6.48	3.55	1.59	18.27	5.6%
	(StDev.)	(4.74 × 10${}^{-5}$)	(1.54 × 10${}^{-5}$)	(4.77 × 10${}^{-5}$)	(5.34 × 10${}^{-4}$)	(8.57 × 10${}^{-5}$)	(5.54 × 10${}^{-4}$)	(3.37 × 10${}^{-5}$)	(2.04 × 10${}^{-5}$)	

**Table 12:** Typical HRV measures after applying cubic spline interpolation and the proposed algorithms for various gap sizes, compared to the HRV measures in the original signal for Participant 2 (NEEDED database).

Signal	Method	RMSSD	SDNN	SDSD	Total Power	LF	HF	SD1	SD2	Mean Error
Original		4.14	13.31	4.16	75.48	17.44	7.80	2.94	18.59	
Gap w = 3	Cubic spline	4.15	13.30	4.16	75.22	16.69	8.12	2.94	18.58	1.2%
	Algorithm 2	4.14	13.29	4.15	74.70	16.22	8.03	2.94	18.56	1.5%
	(StDev.)	(2.97 × 10${}^{-5}$)	(2.77 × 10${}^{-5}$)	(2.99 × 10${}^{-5}$)	(7.89 × 10${}^{-4}$)	(7.44 × 10${}^{-4}$)	(2.86 × 10${}^{-4}$)	(2.11 × 10${}^{-5}$)	(3.86 × 10${}^{-5}$)
Gap w = 5	Cubic spline	4.08	13.15	4.10	72.05	12.34	8.59	2.90	18.36	6.3%
	Algorithm 2	4.10	13.29	4.11	75.31	16.91	8.01	2.91	18.58	1.1%
	(StDev.)	(5.85 × 10${}^{-5}$)	(5.19 × 10${}^{-5}$)	(5.88 × 10${}^{-5}$)	(1.88 × 10${}^{-3}$)	(1.28 × 10${}^{-3}$)	(8.14 × 10${}^{-4}$)	(4.16 × 10${}^{-5}$)	(7.02 × 10${}^{-5}$)
Gap w = 7	Cubic spline	4.09	13.14	4.11	74.18	13.32	9.31	2.90	18.36	6.4%
	Algorithm 2	4.08	13.29	4.10	75.25	16.84	7.91	2.90	18.58	1.1%
	(StDev.)	(7.72 × 10${}^{-5}$)	(8.46 × 10${}^{-5}$)	(7.77 × 10${}^{-5}$)	(2.88 × 10${}^{-3}$)	(2.46 × 10${}^{-3}$)	(1.14 × 10${}^{-3}$)	(5.49 × 10${}^{-5}$)	(1.18 × 10${}^{-4}$)	

**Table 13:** Typical HRV measures after applying cubic spline interpolation and the proposed algorithms for various gap sizes, compared to the HRV measures in the original signal for Participant 3 (NEEDED database).

Signal	Method	RMSSD	SDNN	SDSD	Total Power	LF	HF	SD1	SD2	Mean Error
Original		6.55	23.05	6.48	81.05	16.29	1.70	4.58	32.27	
Gap w = 3	Cubic spline	6.55	23.03	6.48	80.67	16.40	1.87	4.58	32.25	1.4%
	Algorithm 2	6.55	23.06	6.48	81.21	16.26	1.66	4.58	32.28	0.4%
	(StDev.)	(2.04 × 10${}^{-6}$)	(2.55 × 10${}^{-5}$)	(2.09 × 10${}^{-6}$)	(7.05 × 10${}^{-4}$)	(1.06 × 10${}^{-4}$)	(3.51 × 10${}^{-4}$)	(1.48 × 10${}^{-6}$)	(3.65 × 10${}^{-5}$)
Gap w = 5	Cubic spline	6.55	23.03	6.48	80.71	16.34	1.80	4.58	32.25	0.8%
	Algorithm 2	6.56	23.00	6.48	79.90	16.49	2.29	4.58	32.21	4.8%
	(StDev.)	(3.42 × 10${}^{-5}$)	(5.70 × 10${}^{-5}$)	(3.50 × 10${}^{-5}$)	(1.41 × 10${}^{-3}$)	(3.20 × 10${}^{-4}$)	(1.32 × 10${}^{-3}$)	(2.47 × 10${}^{-5}$)	(8.40 × 10${}^{-5}$)
Gap w = 7	Cubic spline	6.55	23.07	6.47	82.05	16.56	1.85	4.58	32.30	1.5%
	Algorithm 2	6.56	23.00	6.49	79.44	16.34	2.20	4.59	32.20	4.0%
	(StDev.)	(5.55 × 10${}^{-5}$)	(1.00 × 10${}^{-4}$)	(5.68 × 10${}^{-5}$)	(4.56 × 10${}^{-3}$)	(5.70 × 10${}^{-4}$)	(1.39 × 10${}^{-3}$)	(4.01 × 10${}^{-5}$)	(1.45 × 10${}^{-4}$)	

**Table 14:** Typical HRV measures after applying cubic spline interpolation and the proposed algorithms for various gap sizes, compared to the HRV measures in the original signal for Participant 1 (PhysioNet database).

Signal	Method	RMSSD	SDNN	SDSD	Total Power	LF	HF	SD1	SD2	Mean Error
Original		32.18	57.26	32.22	2296.47	1295.86	403.44	22.78	77.71	
Gap w = 3	Cubic spline	32.13	57.16	32.16	2285.85	1292.51	400.33	22.74	77.58	0.3%
	Algorithm 2	32.04	56.81	32.07	2244.04	1276.18	386.67	22.68	77.08	1.4%
	(StDev.)	(0.04)	(0.04)	(0.04)	(5.45)	(2.93)	(1.58)	(0.03)	(0.07)
Gap w = 5	Cubic spline	31.58	56.31	31.61	2206.60	1266.42	372.33	22.35	76.43	2.9%
	Algorithm 2	32.46	56.96	32.49	2253.18	1276.37	390.11	22.98	77.21	1.3%
	(StDev.)	(0.17)	(0.17)	(0.17)	(11.04)	(4.62)	(1.92)	(0.12)	(0.21)
Gap w = 7	Cubic spline	31.46	56.23	31.49	2210.85	1270.94	368.61	22.27	76.36	3.1%
	Algorithm 2	33.15	57.28	33.18	2286.60	1299.44	400.71	23.46	77.54	1.3%
	(StDev.)	(0.49)	(0.33)	(0.49)	(11.51)	(10.68)	(9.53)	(0.35)	(0.42)	

**Table 15:** Typical HRV measures after applying cubic spline interpolation and the proposed algorithms for various gap sizes, compared to the HRV measures in the original signal for Participant 2 (PhysioNet database).

Signal	Method	RMSSD	SDNN	SDSD	Total Power	LF	HF	SD1	SD2	Mean Error
Original		27.58	89.45	27.61	1751.53	374.11	253.02	19.52	124.98	
Gap w = 3	Cubic spline	27.48	89.65	27.51	1769.10	386.44	251.03	19.45	125.29	0.8%
	Algorithm 2	28.01	89.19	28.04	1736.91	357.34	265.51	19.82	124.56	1.9%
	(StDev.)	(0.06)	(0.01)	(0.06)	(0.90)	(0.58)	(1.37)	(0.04)	(0.02)
Gap w = 5	Cubic spline	27.37	89.90	27.49	1786.10	395.81	249.53	19.44	125.64	1.4%
	Algorithm 2	27.70	89.23	27.73	1739.86	363.01	260.16	19.61	124.66	1.0%
	(StDev.)	(0.06)	(0.03)	(0.06)	(0.28)	(1.18)	(2.25)	(0.04)	(0.05)
Gap w = 7	Cubic spline	27.44	90.02	27.46	1789.68	395.64	248.16	19.42	125.82	1.6%
	Algorithm 2	27.63	89.37	27.66	1741.50	364.56	255.46	19.56	124.86	0.6%
	(StDev.)	(0.04)	(0.02)	(0.04)	(3.08)	(3.01)	(1.46)	(0.03)	(0.04)	

**Table 16:** Mean error calculated for typical HRV measures after applying cubic spline interpolation and the proposed algorithms for various gap sizes (NEEDED database).

Signal	Method	RMSSD	SDNN	SDSD	Total Power	LF	HF	SD1	SD2
Gap w = 3	Algorithm 2	0.21%	0.07%	0.21%	0.71%	1.60%	3.17%	0.21%	0.09%
	Cubic spline	0.29%	0.09%	0.29%	2.14%	1.97%	8.06%	0.29%	0.11%
Gap w = 5	Algorithm 2	0.35%	0.08%	0.35%	0.93%	1.80%	4.48%	0.35%	0.01%
	Cubic spline	0.38%	0.28%	0.39%	2.78%	4.84%	20.15%	0.39%	0.39%
Gap w = 7	Algorithm 2	0.42%	0.08%	0.43%	0.90%	2.16%	5.60%	0.43%	0.11%
	Cubic spline	0.68%	0.66%	0.68%	6.02%	11.62%	42.56%	19.42068%	0.95%

**Table 17:** Mean error calculated for typical HRV measures after applying cubic spline interpolation and the proposed algorithms for various gap sizes (PhysioNet database).

Signal	Method	RMSSD	SDNN	SDSD	Total Power	LF	HF	SD1	SD2
Gap w = 3	Algorithm 2	0.91%	0.29%	0.91%	0.79%	2.26%	2.37%	0.91%	0.31%
	Cubic spline	0.58%	0.21%	0.58%	0.59%	1.68%	1.23%	0.58%	0.21%
Gap w = 5	Algorithm 2	1.21%	0.40%	1.21%	1.16%	3.87%	3.55%	1.21%	0.42%
	Cubic spline	083%	0.45%	0.83%	1.40%	3.05%	2.84%	0.83%	0.46%
Gap w = 7	Algorithm 2	3.06%	0.36%	3.06%	1.06%	4.28%	4.24%	3.06%	0.31%
	Cubic spline	1.42%	0.85%	1.42%	2.03%	6.13%	4.74%	1.42%	0.89%

**Figure A1:**
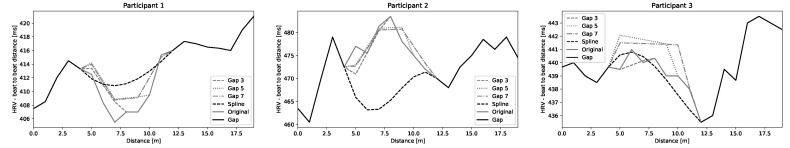
Participants 1–3 with multiple gaps in HRV beat-to-beat duration before filtering and after filtering (NEEDED database).

**Figure A2:**
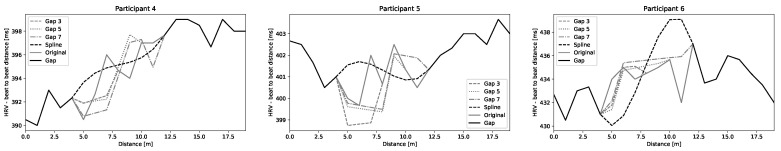
Participants 4–6 with multiple gaps in HRV beat-to-beat duration before filtering and after filtering (NEEDED database).

**Figure A3:**
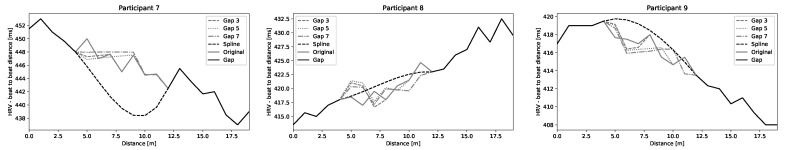
Participants 7–9 with multiple gaps in HRV beat-to-beat duration before filtering and after filtering (NEEDED database).

**Figure A4:**
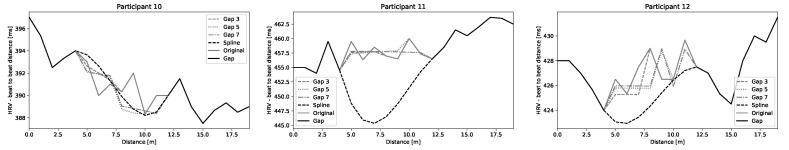
Participants 10–12 with multiple gaps in HRV beat-to-beat duration before filtering and after filtering (NEEDED database).

**Figure A5:**
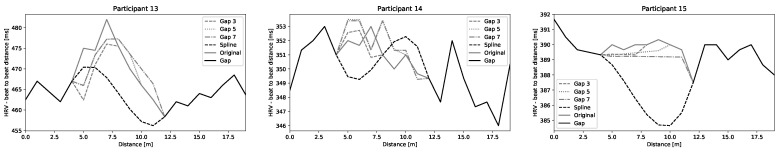
Participants 13–15 with multiple gaps in HRV beat-to-beat duration before filtering and after filtering (NEEDED database).

**Figure A6:**
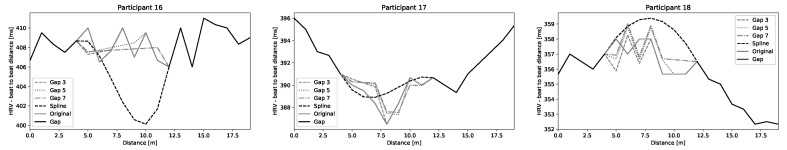
Participants 16–18 with multiple gaps in HRV beat-to-beat duration before filtering and after filtering (NEEDED database).

**Figure A7:**
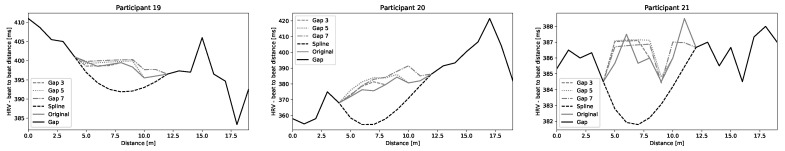
Participants 19–21 with multiple gaps in HRV beat-to-beat duration before filtering and after filtering (NEEDED database).

**Figure A8:**
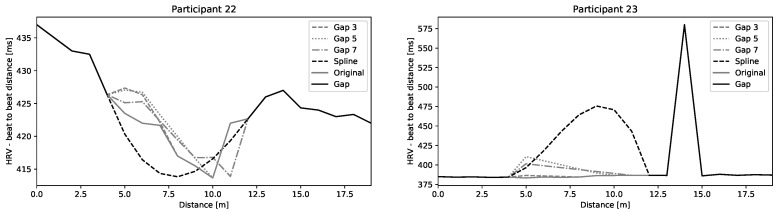
Participants 22 and 23 with multiple gaps in HRV beat-to-beat duration before filtering and after filtering (NEEDED database).

## Appendix A

Figure A1, Figure A2, Figure A3, Figure A4, Figure A5, Figure A6, Figure A7 and Figure A8 present results of Algorithm 2 for participants of NEEDED project.

## Appendix B

Figure A9, Figure A10, Figure A11, Figure A12, Figure A13, Figure A14, Figure A15, Figure A16, Figure A17, Figure A18, Figure A19, Figure A20 and Figure A21 present results of 50 iterations of Algorithm 2 applied to signals from PhysioNet database.
